# The Effect of Lexical Cohort Size Is Independent of Semantic Context Effects in a Picture–Word Interference Task: A Combined ERP and sLORETA Study

**DOI:** 10.3389/fnhum.2019.00439

**Published:** 2019-12-20

**Authors:** Mingkun Ouyang, Xiao Cai, Qingfang Zhang

**Affiliations:** Department of Psychology, Renmin University of China, Beijing, China

**Keywords:** semantic interference, semantic facilitation, lexical cohort size, speech production, PWI

## Abstract

Lexical cohort size is known to play an important role in the magnitude of semantic interference during picture naming in continuous and blocking naming tasks. Nevertheless, whether and how lexical cohort size influences semantic context effects in a picture–word interference (PWI) task remains unclear. To address this issue, participants were required to name pictures, which were paired with both semantically related and unrelated distractors, from both large and small lexical cohorts while electroencephalogram (EEG) signals were recorded. Behavior results showed a semantic interference effect but no interaction between semantic relatedness and lexical cohort size in naming latencies. ERPs and correlation analyses revealed that semantic interference effects occurred at the lexical level in the time windows of 200–400 and 400–600 ms, and lexical cohort size effects occurred at the conceptual level in the time window of 100–200 ms and at the lexical level in the time windows of 200–400 ms. Critically, no interaction between two variables was found, reflecting that lexical cohort size is independent of semantic interference for categorical relations in the PWI. sLORETA results found stronger brain activations for large lexical cohorts at the left superior temporal gyrus and inferior frontal gyrus in the time interval of 250–300 ms, which may relate to lexical selection and self-monitoring. Our findings provide evidence for the swinging lexical network rather than the response exclusion hypothesis in spoken production.

## Introduction

A recent debate in language production literature analyzes how speakers retrieve words from the mental lexicon. The picture–word interference (hereafter PWI) paradigm, a variant of the Stroop task, has been broadly used to investigate the word retrievals within the field of speech production (Mahon et al., 2007; Dell’Acqua et al., 2010; Zhu et al., 2015, 2016; Wong et al., 2017). In this paradigm, speakers are instructed to name pictures while ignoring written distractor words superimposed onto the pictures. A typical finding using the PWI task is the semantic interference effect, which means that categorically related distractors (e.g., “bus”) slow down picture naming (e.g., “car”) relative to unrelated distractors (e.g., “pen”) (Abdel Rahman and Melinger, 2007; de Zubicaray et al., 2009; Aristei et al., 2011). Intriguingly, not all semantic contexts lead to interference; instead, associative or part–whole relations between the target and distractor facilitate picture naming (Costa et al., 2005; Abdel Rahman and Melinger, 2007).

Semantic facilitation has been envisaged as the semantic priming of the target concept (Finkbeiner and Caramazza, 2006; Mahon et al., 2007; Abdel Rahman and Melinger, 2009a,b), while semantic interference reflects the lexical selection competition among co-activated semantically related lexical representations during lemma retrieval (Roelofs, 1992, 2018; Starreveld and La Heij, 1996; Levelt et al., 1999; Abdel Rahman and Melinger, 2009a,b) or arises from the response exclusion difficulty given that semantically related distractors are more difficult to exclude from the response buffer than unrelated ones due to response relevance (Finkbeiner and Caramazza, 2006; Mahon et al., 2007).

In order to interpret both semantic facilitation and interference effects in a theoretical framework, Abdel Rahman and Melinger (2009a,b) proposed a variant of the lexical competition model, namely the swinging lexical network account (SLN). This account assumes that the semantic facilitation effect mirrors the semantic priming of the target concept functioning at the conceptual level (see also Costa et al., 2005; Finkbeiner and Caramazza, 2006; Mahon et al., 2007), and that semantic interference arises from strong competition among a lexical cohort that is co-activated with the target. The polarity of the semantic context effect then depends on a trade-off between conceptual priming (semantic facilitation) and lexical competition (semantic interference). According to the SLN, the interference-dominant semantic effect results from the activation of a lexical cohort, which consists of a set of semantically interrelated lexical representations. Theoretically, the magnitude of interference is associated with the size of the lexical cohort, with larger sizes inducing stronger interference (Abdel Rahman and Melinger, 2009b, 2019; Melinger and Abdel Rahman, 2013; Rose and Abdel Rahman, 2017).

### The Influence of Lexical Cohort Size on Semantic Context Effects

Studies have shown that the lexical cohort size is greatly determined by the number of newly named members in a given context, and it further exerts an important influence on the magnitude of semantic facilitation and interference (Abdel Rahman and Melinger, 2009a,b, 2011). For example, Rose and Abdel Rahman (2016) reported that the size of the lexical cohort systematically increased in a continuous naming task, in which different members of a given thematic context were consecutively presented to participants. Similar results were also observed in a cyclic blocking naming task, in which participants were required to name a series of pictures belonging to a semantic category or sharing semantic features (Belke and Stielow, 2013). More importantly, results in these tasks showed that the interference effect increased linearly with the size of the lexical cohort (Costa et al., 2009; Abdel Rahman and Melinger, 2011; Belke and Stielow, 2013; Schnur, 2014). All these studies suggested that semantic context effects are modulated by lexical cohort size, which are highly dynamic and adaptive in a special context (Abdel Rahman and Melinger, 2009a,b, 2011).

Various naming tasks, however, differ in their capacity to induce a lexical cohort. Both continuous and cyclic blocking naming tasks investigate lexical cohort size by manipulating the number of newly named members. In this way, participants could be aware of the categorically related members. The PWI task is incapable of directly measuring the lexical cohort size as objects belonging to a given category are not presented or named previously. In this way, participants cannot be aware of the categorically related members. Therefore, it is important to pay attention to the PWI task to uncover the mechanisms underlying how lexical cohort size influences semantic context effects. Indeed, in the PWI task, the semantic relations between target and distractor (categorically or associatively related) provide distinct constraints on the size of the lexical cohort, which accordingly alters the semantic context effects (Roelofs, 1992; Levelt et al., 1999). Specifically, when the target (e.g., car) and distractor (e.g., bus) are exemplars of the same category (i.e., vehicle), they automatically spread converging activation to other exemplars (e.g., train and airplane) for the shared categorically related features (Abdel Rahman and Melinger, 2009b). In other words, all exemplars in a given category form a large size of lexical cohort (Abdel Rahman and Melinger, 2007; Rabovsky et al., 2016). In contrast, when the target (e.g., bee) and distractor (e.g., honey) are associatively related but belong to different categories, activation from them does not converge onto other related concepts. No shared nodes between target and distractor thus induce a small size of lexical cohort in the PWI task.

The literature has mainly focused on how lexical cohort size influences the nature of semantic context effects (i.e., facilitation or interference) (Alario et al., 2000; Abdel Rahman and Melinger, 2007), but it remains an open question whether and how the magnitude of the semantic context effect is moderated by lexical cohort size induced by categorical relations. To address this issue, a number of studies using the PWI task have manipulated the semantic distance between target and distractor. The rationale is that a semantically distant distractor activates a large size of lexical cohort with more members, whereas a close one activates a small size of lexical cohort with fewer members. In line with this notion, Mahon et al. (2007) reported a stronger interference effect for semantically distant distractors (e.g., target: horse, distractor: whale) than semantically close distractors (e.g., target: horse, distractor: zebra). However, growing evidence has shown a contrasting finding that stronger interference was observed in close distractors as opposed to distant ones (Vigliocco et al., 2002; Vieth et al., 2014; Rose et al., 2019). Besides, a null effect for lexical cohort size has been reported as well (Hutson and Damian, 2014).

These mixed findings may originate from inconsistent measures of lexical cohort size across studies. For example, some studies measured lexical cohort size via semantic distance based on isolated features shared by target and distractor, even though they did not share categorically related features (Vigliocco et al., 2002; Hutson and Damian, 2014), while others operationally defined lexical cohort size via semantic similarity ratings based on shared categorical features (Mahon et al., 2007; Vieth et al., 2014). To avoid these inconsistencies, Rose et al. (2019) manipulated lexical cohort size within taxonomic hierarchies. Specifically, when the target (e.g., parrot) and distractor (e.g., camel) belonged to a superordinate category (e.g., animals) but different basic categories (birds vs. mammals), a large lexical cohort size was induced due to fewer shared semantic features; but when they (e.g., target: parrot, distractor: owl) were members of a basic category (e.g., birds), a small lexical cohort size was induced due to many shared features. Contrary to the prediction derived from the SLN, they found that the semantic interference effect was stronger for small lexical cohorts compared with large ones. This finding was extrapolated from the presence of a stronger activation strength of lexical items in small lexical cohorts than in large ones (Rose et al., 2019). There is evidence to suggest that the activation strength of lexical items is largely determined by the number of semantic features overlapping among concepts (Roelofs, 1992; Vigliocco et al., 2002; Vieth et al., 2014). Based on this argument, exemplars (i.e., competitors) sharing many semantic features in a basic level category could be more active compared with those sharing fewer features from a superordinate category. In other words, competitors in small cohorts may be strongly activated and then produce fiercer lexical competition than those in large cohorts (Abdel Rahman and Melinger, 2019; Rose et al., 2019).

Similarly, Bormann (2011, Experiment 3) manipulated lexical cohort size in terms of the semantic category size in a PWI task. Unlike Rose et al. (2019), they selected target–distractor pairings only from basic-level categories, and these selected categories were divided into large and small categories based on a seven-point rating task in which participants judged whether there existed “many,” “some,” or “hardly any” competitors to targets. Results of this study revealed no difference in the magnitude of semantic interference between different sizes of lexical cohort. These findings are of great import to understanding the role of lexical cohort size in the semantic interference as the strength of lexical activation between large and small sizes of lexical cohorts was well controlled by employing only the basic categories, unlike the study by Rose et al. (2019), which involved both basic and superordinate categories. However, it is unknown whether a self-reported seven-point scale is a valid measure of lexical cohort size. Given the methodological limitation, therefore, it is still insufficient to conclude that lexical cohort size plays little role in the magnitude of semantic context effects in a PWI task. To avoid this problem, the present study manipulated the lexical cohort size (i.e., category size) based on the number of exemplars in the basic-level categories. Besides, considering the null behavioral effect in the previous study, we further tested whether the lexical cohort size effect can be detected using sensitive electrophysiological (EEG) measures. Overall, the present study aimed to investigate whether and how lexical cohort size influenced the semantic context effects in the PWI task using the EEG technique.

### The Temporal Courses and Neural Correlates of Semantic Context Effects

Studies regarding speech production have shown that stimulus-aligned Event-Related Potentials (ERPs) could be well employed without significant contamination to the signals before articulation (Dell’Acqua et al., 2010; Aristei et al., 2011; Janssen et al., 2015; Rose and Abdel Rahman, 2017). Since articulation-related artifacts may emerge around 150 ms (Fargier et al., 2017) or 300 ms before voice onset (Ouyang et al., 2016), it is effective to analyze the semantic context effects occurring in the stage of word planning rather than articulatory buffering (Roelofs and Piai, 2015).

While studies on the semantic facilitation effect are restricted to the context of associative relations, categorical relations also induce facilitation effects (Mahon et al., 2007). As for the time course of the semantic facilitation effect, previous researchers found it to be located in the area of conceptual preparation (Finkbeiner and Caramazza, 2006; Mahon et al., 2007; Abdel Rahman and Melinger, 2009a,b). Thus, according to the two meta-analysis studies on speech production, the semantic facilitation effect should take place in the time window of 0–200 ms after picture onset (Indefrey and Levelt, 2004; Indefrey, 2011).

Many EEG studies have attempted to gain insight into the time course of the semantic interference effect, and most of these have located it at the lexical-semantic level, in line with the assumption of competitive models. Based on the meta-analysis studies, lemma retrieval starts approximately from 200 ms and completes about 350 ms post picture onset (Indefrey and Levelt, 2004; Indefrey, 2011). However, there is no general agreement on the onset latency or duration of the semantic interference effect, and this is partly due to the experimental tasks and materials adopted. For instances, evidence has shown that semantic interference emerges either between 250–400 ms (Rose and Abdel Rahman, 2017) or 200–380 ms (Costa et al., 2009) in the continuous naming task, an interval of 250–300 ms in a semantic blocking task, and in the time window of 250–370 ms (Piai et al., 2012), or at around 320 ms post stimulus onset, in a PWI task (Dell’Acqua et al., 2010). Thus, it is reasonable to observe a relative late time window (i.e., around 200–400 ms) associated with lemma selection.

Regarding the brain regions involved in the lexical cohort size effect, previous fMRI studies found increased activation for target names from a large lexical cohort compared with a small one in the posterior superior temporal gyrus (pSTG), supramarginal gyrus (SMG), inferior frontal gyrus (IFG) (Mirman and Graziano, 2013), as well as the superior temporal sulcus (STS) (McGuire et al., 1996). The higher activation may have been reflective of a mechanism of lexical activation or lexical selection among competing alternatives. Besides, evidence has indicated that IFG plays a crucial role in cognitive control, such as verbal self-monitoring involved in lexical selection when semantic interference occurs (Schnur et al., 2009; Dhooge and Hartsuiker, 2012; Mirman and Graziano, 2013). Thus, it is reasonable to postulate that these brain regions would be involved in the lexical cohort size effect and semantic context effects in picture naming using the PWI task.

### The Current Study

The present study aimed to investigate the effect of lexical cohort size on the magnitude of semantic context effects during picture naming using a PWI task combined with EEG and standardized low-resolution brain electromagnetic tomography (sLORETA) techniques. Specifically, we were interested in the temporal courses of lexical cohort size and categorically semantic relatedness: their interaction and how they influence spoken word production. According to the SLN (Abdel Rahman and Melinger, 2009a,b), we predicted a larger semantic interference for larger cohorts in comparison with small cohorts. For the ERP results, we expected both lexical cohort size and semantic relatedness, and their interaction, to influence early conceptual and late lexical selection processes. Based on the SLN’s assumption, we also expected to find an early semantic facilitation effect while a relatively late semantic interference effect.

In addition to ERPs, a method of sLORETA was applied to locate brain regions implicated in the generators of effects of semantic interference and lexical cohort size (Pascual-Marqui, 2002). This method employed the smoothest spatial source distribution by minimizing the Laplacian of the weighted sources without *a priori* assumption about a predefined number of activated brain regions (Pascual-Marqui, 2002), and it thus provided a more open solution to the EEG inverse problem (see below for details). Several studies have confirmed the validation of the localization accuracy by comparing sLORETA with neuroimaging techniques, such as fMRI (Pizzagalli et al., 2004). During a typical picture-naming process, the lexical cohort size effect lasts only 200 ms (Costa et al., 2009), so it is hard to figure out the neural correlates in fMRI techniques with low temporal resolution. For this reason, sLORETA analysis could shed new insight into the neural correlates underlying the lexical cohort size and semantic interference effects. Based on the previous findings reviewed above, we hypothesize that these effects would be strongly associated with brain regions like the temporal gyrus and IFG.

## Materials and Methods

### Participants

Thirty-one undergraduates participated in the experiment (13 males, aged from 18 to 32 years). All participants were native Mandarin speakers and neurologically healthy, and they had normal or corrected-to-normal vision and normal hearing. All participants gave their informed consent prior to the experiment and were paid for their participation. This study was approved by the ethics board of the Renmin University of China.

### Stimuli and Design

Forty-eight target pictures with names corresponding to disyllabic or trisyllabic Chinese characters were selected from a standardized picture database (Zhang and Yang, 2003). The typical picture names belonged to eight categories: mammals, birds, fruits, vegetables, body organ, instruments, furniture, and appliances. The lexical cohort size was operationally defined based on the number of exemplars in a given category. The number of exemplars for a specific category was adopted from a database by Fang and Zhang (2013). A large lexical cohort size refers to a category (i.e., mammals, birds, fruits, and vegetables) whose total number of exemplars is more than 40, while a small lexical cohort size refers to a category (i.e., body organ, instruments, furniture, and appliances) whose total number of exemplars is less than 30. It has been proposed that the member of a small lexical cohort size category is less than 30 exemplars in the literature (i.e., Karasawa and Brewer, 1996; Forster, 2004; Fang and Zhang, 2013). Each category comprised of six pictures in the experiment. An independent *t*-test revealed a significant difference in the number of exemplars between large (*M* = 50.25, *SD* = 8.66) and small (*M* = 25.75, *SD* = 3.20) lexical cohorts, *t*(6) = 5.31, *p* = 0.002.

Pictures from large and small categories were controlled for variables like image variability, image agreement, concept familiarity, visual complexity, subjective frequency, name agreement, and naming latency (*t*s < 1, *p*s > 0.05 in all conditions) (see Table 1). Each picture was paired with a semantically related distractor that was a highly typical member of its corresponding category and an unrelated distractor with no semantic, phonological, and orthographic relationship to the target name. Statistical analyses showed no significant differences between related and unrelated items regarding the number of strokes, printed lexical frequency, concept familiarity, or concreteness (*t*s < 1, *p*s > 0.05 in all conditions) (see Table 2). The materials used are presented in Supplementary Appendix A.

**TABLE 1:** Summary of statistics for the target pictures used in the experiment.

**Picture properties**	**Large lexical cohort size (*M* ± *SD*)**	**Small lexical cohort size (*M* ± *SD*)**
Image variability	3.00 ± 0.21	2.93 ± 0.29
Image agreement	4.07 ± 0.31	3.92 ± 0.36
Concept familiarity	4.52 ± 0.29	4.39 ± 0.44
Visual complexity	2.94 ± 0.76	2.76 ± 0.65
Subjective frequency	2.84 ± 0.55	3.00 ± 0.69
Name agreement	0.99 ± 0.71	1.14 ± 0.49
Naming latency (ms)	981.61 ± 199.38	1047.25 ± 201.16

**TABLE 2:** Summary of statistics of the context words used in the experiment.

**Lexical properties**	**Large lexical cohort size**		**Small lexical cohort size**	
		
	**Semantically related**	**Semantically unrelated**	**Semantically related**	**Semantically unrelated**
Semantic similarity	4.89	1.26	4.54	1.89
Concreteness	4.88	4.60	4.56	4.55
Concept familiarity	4.72	4.61	4.40	4.49
Strokes	16.46	16.63	16.75	16.04
Lexical frequency	5.87	5.88	5.81	5.83

The experimental design included the lexical cohort size (large vs. small) and semantic relatedness (related vs. unrelated) as within-participant factors. Within an experimental block, participants named each picture twice (one in related and the other in unrelated) for a total of 96 trials. The block was repeated twice due to the limitation of available items, and the entire experiment thus consisted of 192 trials. The order of items within each block was pseudo-randomized with the constrain that a particular category did not repeat on consecutive trials. A new sequence was generated for each participant and each block. Note that a design in which each target is presented and named multiple times is quite common in spoken word production and usually considered to be unproblematic (i.e., Zhu et al., 2015; Qu et al., 2016; Zhang and Damian, 2019). Nevertheless, in the latency analysis reported below, we included “repetition” as an addition factor to check for potential effects of multiple target presentation.

In order to match the effect of semantic relatedness between targets and distractors, we assessed the degree of semantic relatedness between targets and categorical semantic distractors by recruiting 20 native Chinese speakers (five males, aged from 19 to 28 years) who did not take part in the formal experiment. Target picture names were paired with their corresponding semantically related and unrelated distractor words. The word pairs were presented in a random order, and pictures from the same category were avoided in consecutive trials. The word pairs were rated on a five-point scale, with five indicating that word pairs were highly semantically related and one indicating that word pairs were semantically unrelated.

For a large cohort size, the average degree of semantic relatedness was 4.89 (*SD* = 0.48) between semantically related distractors and target names, and it was 1.26 (*SD* = 0.53) between unrelated distractors and target names across subjects. A aired *t* test indicated a significant difference between two semantic relatedness degrees, *t*(19) = 42.74, *p* < 0.001. For a small lexical cohort size, the average degree of semantic relatedness was 4.54 (*SD* = 0.59) between semantically related distractors and target names, and it was 1.89 (*SD* = 0.54) between unrelated distractors and target names across subjects. A paired *t* test indicated a significant difference between two semantic relatedness degrees, *t*(19) = 40.21, *p* < 0.001. Importantly, there was no significant difference in the semantic relatedness between two semantically related distractor words, *t*(19) = 1.03, *p* = 0.16, suggesting that the activation level of semantically related distractors from large and small categories could be the same.

#### Procedure and Apparatus

Participants were tested individually in front of a computer screen in a soundproof lab. Participants were first required to familiarize themselves with target pictures by viewing each picture for 3,000 ms with the correct name presented below. Participants were then informed that their task was to name the target as quickly and accurately as possible while ignoring the distractor. Participants received 12 warm-up trials to familiarize themselves with the procedure before the formal experiment.

Each trial strictly involved the following sequence: a fixation point (+) presented in the middle of the screen for 500 ms followed by a blank screen for 500 ms. Subsequently, the target picture plus the distractor word were presented simultaneously, and an inter-trial interval of approximately 2,000 ms concluded each trial. Targets would disappear when participants initiated a voice response. There was a 2 min break between two blocks, and the next block started after participants indicated that they were ready to continue. The entire experiment took about 1 h in total.

The experiment was programed in E-Prime Professional Software (Version 2.1) using a fast Lenovo compatible PC. The stimuli were presented on a 19-inch LCD monitor with a refresh rate of 100 Hz. The naming latencies were measured from picture onset using a voice key, which was connected to the computer via a PST Serial Response Box.

### EEG Acquisition and Analysis

Continuous EEG signals were recorded by 64 electrodes in an elastic cap using a Neuroscan system (SymAmps 2.0). The electrode sites followed the extended 10–20 system with the left mastoid electrode as reference. Electrophysiological signals were amplified with a band-pass filter of 0.05–100 Hz, and they were digitized at a sampling rate of 500 Hz. Horizontal EOG (HEOG) was recorded bipolarly from outer canthi of both eyes. Vertical EOG (VEOG) was measured from two electrodes, one below and the other one above the left eye. All electrode impedances were kept below 5 kΩ.

The package Neuroscan 4.3 was used in the ERP data analysis. In the offline analysis, the EEG data were re-referenced to the average of both mastoids and filtered using a bandpass of 0.1–30 Hz. The data were segmented from 200 ms before to 600 ms after the onset of the pictures, with baseline correction from -200 to 0 ms preceding picture onset. Epochs containing an artifact signal below/above ±100 μV were rejected. Prior to offline averaging, all single-trial waveforms were screened for eye movements, electrode drifting, amplifier blocking, and artifacts.

The articulation-related motion artifact (i.e., muscular artifacts) could impact the EEG signal of interest. A typical way to deal with artifacts is to avoid analyzing the signal too close to the articulation. The articulation buffer is estimated to be reached no earlier than 145 ms before articulation onset (Indefrey, 2011). However, given that the averaged naming latency is usually longer than 600 ms, it is plausible to assume that the onset of articulation would occur after 455 ms. Some researchers argue that rescaling is required regarding the stage durations of all processing stages if the average naming time is different from 600 ms (Roelofs and Shitova, 2016; Wong et al., 2017). For example, Dhooge et al. (2013) observed a mean picture-naming time of 780 ms, and the onset of articulation in Dhooge et al. was estimated to occur around 635 ms post pictures by linear rescaling. Ouyang et al. (2016) provided evidence that articulation-related artifacts could start up to 300 ms or more prior to articulation. Fargier et al. (2017) found an earlier component onset around 150 ms before vocal onset when comparing the different movements of voice sounds. Wong et al. (2017) observed an averaged naming time of 832 ms, and the onset of articulation was estimated to occur around 687 ms after pictures onset. Given that the average naming latency was 900 ms in the present study, we selected an epoch from 200 ms before to 600 ms after pictures onset.

Nine regions of interest (ROIs) were selected for statistical analysis, i.e., left-anterior (pooled F3, F5, and FC3), mid-anterior (Fz), right-anterior (pooled F4, F6, and FC4), left-central (pooled C3, C5, and CP3), mid-central (Cz), right-central (pooled C4, C6, and CP4), left- posterior (pooled P3, P5, and PO3), mid-posterior (Pz), and right-posterior (pooled P4, P6, and PO4) regions, and the voltage of each ROI was the mean amplitude of the member electrodes (Zhu et al., 2015). Mean amplitudes were calculated for each participant and each condition in the four time windows, 0–100, 100–200, 200–400, and 400–600 ms, which roughly correspond to early (0–100 ms) and late conceptual preparation (100–200 ms), lexical selection (200–400 ms), and articulation preparation (400–600 ms) processes estimated by a meta-analysis (Indefrey, 2011). In addition, we determined these time windows by visually inspecting the averaged ERP waveforms across the conditions (see Luck and Gaspelin, 2017 for a similar approach). A repeated measure analysis of variance (ANOVA) was performed on the amplitude means with the factors of semantic relatedness, lexical cohort size, ROIs, and “repetition” conducted separately for each time window. A Greenhouse–Geisser correction was applied where appropriate, and all the results relied on a 5% significance level.

### Source Estimate

To identify possible differences in the brain electrical activity between lexical cohort sizes (large vs. small) and between semantic relatedness (related vs. unrelated), we calculated sLORETA images for each participant and then calculated the sLORETA images across all participants for each lexical cohort size and semantic relatedness with a time step of 50 ms from picture onset (0 ms) to 600 ms post picture presentation (Wieser et al., 2010). Furthermore, the sLORETA images were compared across conditions to determine the significant differences (corrected, *p* < 0.05) in the brain electrical activity, using the log-*F*-ratio statistic with sLORETA-built-in voxel-wise randomization tests (5,000 random permutations) (Nichols and Holmes, 2002).

## Results

### Behavioral Results

An incorrect response and response time (RT) shorter than 300 ms or longer than 2,000 ms (4.66%), and those deviating by more than two standard deviations from a participant’s mean (5.08%), were removed from all analyses. Table 3 exhibits the mean latencies and error percentages, and they are presented by semantic relatedness and lexical cohort size.

**TABLE 3:** Mean naming latency (RT, in ms), mean error percentages (%), standard deviations, and semantic interference effects (SI in ms).

	**Large lexical cohort size**		**Small lexical cohort size**	
		
	**RT (*SD*)**	**Error (*SD*)**	**RT (*SD*)**	**Error (*SD*)**
Semantically related	906.60 (189.11)	0.08 (0.03)	923.67 (192.17)	0.17 (0.04)
Semantically unrelated	888.22 (184.18)	0.16 (0.04)	911.27 (187.34)	0.07 (0.02)
SI effect	18^∗^		12^∗^	

*^∗^*p* < 0.05. The asterisk represents the latency in the semantic-related condition, which significantly differs from the unrelated condition.*

We used the *lmer* program of the *lme4* package (Bates et al., 2014) in R software (R Development Core Team, 2009) to estimate fixed and random effects. The data (i.e., RT and the percentage of error response) were analyzed using a linear mixed-effects model with semantic relatedness, lexical cohort size, and repetition as the fixed factors with participants and items as the random factors. Models used restricted maximum likelihood estimation to find the optimal parameter estimation of the best-fitting model to the observed data. The best-fitting model was defined as the most adjustment model that significantly improved the variance estimation over previous models. Model fitting mainly includes three steps: first, specifying a model (i.e., null model) that included only random factors (participants and items); second, enriching the null model by adding fixed factors (i.e., semantic relatedness, lexical cohort size, and repetition) one by one and adding three two-way interactions among three factors and one three-way interaction among factors one by one to previous models. Third, comparing the newly established model to a previous model using the chi-square test. If adding a fixed factor or an interaction among factors to an existing model did not significantly improve the variance estimation, then the current model is the best fitting model.

For response latencies, the best-fitting model only included factors of semantic relatedness, lexical cohort size, and repetition (see Table 4). Adding the two-way interactions between semantic relatedness and lexical cohort size, χ2 (1, 4757) = 0.0015, *p* = 0.97, semantic relatedness and repetition, χ2 (1, 4757) = 0.50, *p* = 0.48, lexical cohort size and repetition, χ2 (1, 4757) = 0.16, *p* = 0.69, and the three-way interaction between semantic relatedness, lexical cohort size, and repetition, χ2 (1, 4757) = 0.78, *p* = 0.94 did not significantly improve the fit^1^.

**TABLE 4:** LMM estimates of fixed effects for response latencies in picture naming.

**Fixed effects**	**Measure**		
	
	**Estimate**	**Std. Error**	***t*-value**
(Intercept)	932.026	21.504	43.341^∗∗∗^
LCS2	31.989	12.376	2.585^∗^
SR2	–24.945	13.650	−1.827^†^
Repetition2	–34.167	9.252	–3.693^∗∗∗^

*LCS2, Small lexical cohort size; SR2, semantically unrelated; Repetition2, Block2. ^∗∗∗^*p* < 0.001, ^∗^*p* < 0.05, ^†^0.05 < *p* < 0.1.*

For the percentage of error response, the best-fitting model only included random factors of participants and items. Adding fixed factors (semantic relatedness, lexical cohort size, and repetition), three two-way interactions, and one three-way interaction did not significantly improve the fit of null model (all *p*s > 0.21).

### Electrophysiological Results

Two participants were excluded because of excessive artifacts, and subsequent analyses were carried out on the remaining 29 participants.

The repeated measures ANOVA on the averaged amplitudes was conducted separately for four consecutive time windows; the variables semantic relatedness and lexical cohort size were factorially crossed, and it also included the variables of ROI and repetition. Similar to the previous ERP studies using a PWI task, the ERP analysis in the present study covered the period of the first 600 ms post-target onset (Zhu et al., 2015; Wong et al., 2017), which was assumed to be sufficient to cover the time window of word planning (Indefrey and Levelt, 2004; Indefrey, 2011). Semantic relatedness was detected to have a significant effect during the 200–400 and 400–600 ms time windows, and the same was found in the lexical cohort size in the 100–200 and 200–400 ms time windows. Notably, the two-way interaction between lexical cohort size and semantic relatedness was not significant in all time windows. Similar results were obtained for the interactions between repetition, semantic relatedness, and lexical cohort size (see Table 5).

**TABLE 5:** Results of the repeated measures ANOVAs for the factors semantic relatedness (SR), lexical cohort size (LCS), repetition, and ROI.

**Sources of variation (df1, df2)**		**Time windows**			
		
		**0–100 ms**	**100–200 ms**	**200–400 ms**	**400–600 ms**
SR(1,28)	*F*	0.058	0.249	6.787^∗^	4.394^∗^
	η*_*p*_*^2^	0.002	0.009	0.195	0.136
LCS(1,28)	*F*	0.026	5.319^∗^	5.191^∗^	0.779
	η*_*p*_*^2^	0.001	0.160	0.156	0.027
Repetition	*F*	1.395	0.141	0.429	0.259
	η*_*p*_*^2^	0.047	0.005	0.015	0.009
ROI(8,224)	*F*	16.358^∗∗∗^	1.830	15.726^∗∗∗^	16.217^∗∗∗^
	η*_*p*_*^2^	0.369	0.061	0.360	0.367
SR × LCS(1,28)	*F*	0.368	2.712	1.113	1.285
	η*_*p*_*^2^	0.013	0.088	0.038	0.044
SR × Repetition(1,28)	*F*	0.008	0.056	0.058	0.758
	η*_*p*_*^2^	0.001	0.002	0.002	0.026
SR × ROI(8,224)	*F*	1.351	0.716	1.238	1.512
	η*_*p*_^2^*	0.046	0.025	0.042	0.051
LCS × Repetition(1,224)	*F*	3.502	1.617	0.576	2.502
	η*_*p*_*^2^	0.111	0.079	0.454	0.082
LCS × ROI(8,224)	*F*	0.874	0.955	2.945^∗^	1.414
	η*_*p*_*^2^	0.030	0.033	0.095	0.048
Repetition × ROI(8,224)	*F*	0.801	0.923	0.710	0.849
	η*_*p*_*^2^	0.028	0.032	0.025	0.029
SR × LCS × Repetition(1,224)	*F*	0.237	2.172	4.079^†^	4.005^†^
	η*_*p*_*^2^	0.008	0.072	0.127	0.125
SR × LCS × ROI(8,224)	*F*	0.213	0.584	0.512	0.324
	η*_*p*_*^2^	0.008	0.020	0.018	0.011
SR × Repetition × ROI(8,224)	*F*	0.693	1.147	0.601	0.729
	η*_*p*_^2^*	0.024	0.039	0.021	0.025
LCS × Repetition × ROI(8,224)	*F*	1.452	2.090	1.787	1.011
	η*_*p*_*^2^	0.049	0.069	0.060	0.035
SR × LCS × Repetition × ROI(8,224)	*F*	1.342	0.879	1.589	1.709
	η*_*p*_*^2^	0.046	0.030	0.054	0.058

*^∗∗∗^*p* < 0.001, ^∗^*p* < 0.05, ^†^0.05 < *p* < 0.1.*

**FIGURE 1:**
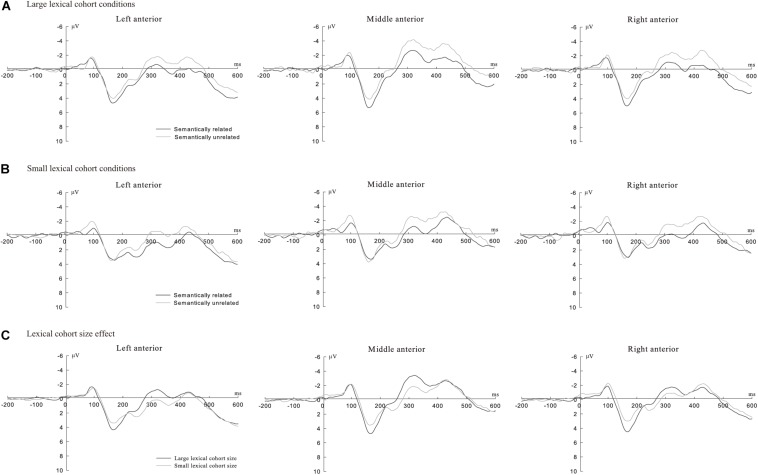
**(A)** Grand average ERPs in semantically related (black line) and unrelated condition (gray line) under large lexical cohort conditions in anterior regions (Upper panel); **(B)** grand average ERPs in semantically related (black line) and unrelated condition (gray line) under small lexical cohort conditions in anterior regions (Middle panel); and **(C)** grand average ERPs in large (black line) and small lexical cohort size (gray line) conditions in anterior regions (lower panel).

**FIGURE 2:**
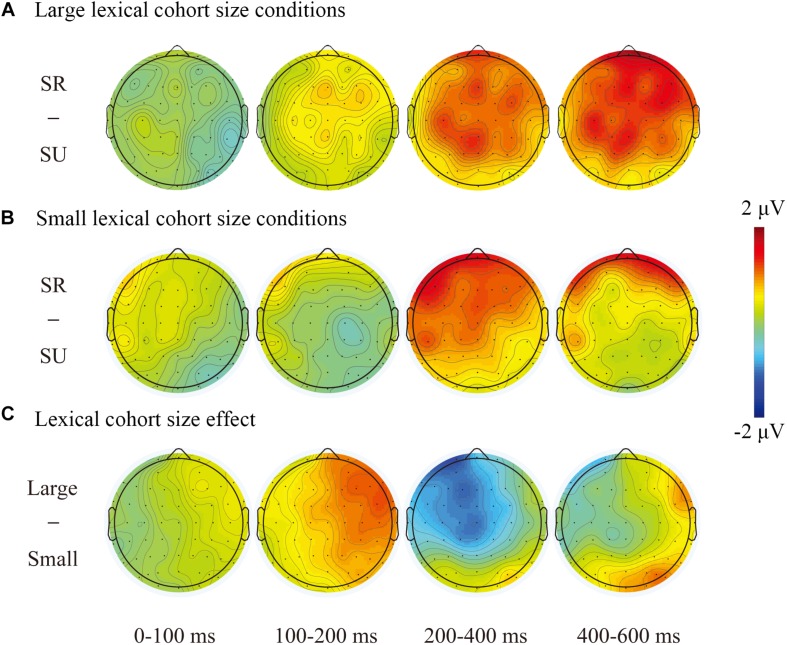
**(A)** Topographical maps of the semantic interference effect (semantically related minus unrelated) under large lexical cohort conditions (Upper panel); **(B)** topographical maps of the semantic interference effect (semantically related minus unrelated) under small lexical cohort conditions (Middle panel); and **(C)** topographical maps of the lexical cohort size effect (large minus small) (lower panel).

Additionally, we observed a more negative-going waveform in the semantically unrelated than that in the semantically related (see Figures 1A, 2A for large cohort size condition and Figures 1B, 2B for small cohort size) and a more negative waveform in the large cohort size than that in the small cohort size (see Figures 1C, 2C).

### Correlation Analysis Between ERP Amplitudes and Naming Latencies

**FIGURE 3:**
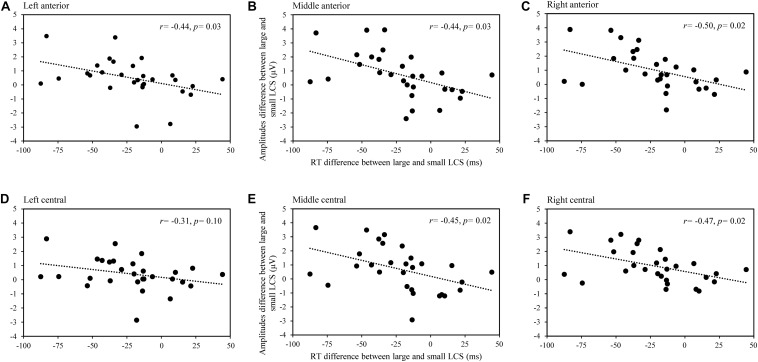
The scatterplot for the correlation between RT (large subtracting small lexical cohort size conditions) and the mean amplitudes (large subtracting small lexical cohort size conditions) in the time window of 100–200 ms in left-anterior **(A)**, mid-anterior **(B)**, right-anterior **(C)**, left-central **(D)**, mid-central **(E)**, and right-central **(F)** regions.

To determine the underlying mechanism of ERP for the lexical cohort size effect, we performed a Pearson correlation analysis between the difference of naming latencies (large minus small) and difference of mean amplitude (large minus small) for large and small lexical cohort conditions across nine ROIs in the time windows of 100–200 and 200–400 ms, respectively. The *FDR* correction was applied to the statistics in different time windows. Analysis showed significant negative correlations in the frontal and central regions in the time window of 100–200 ms, including the left-anterior, *r*(29) = -0.441, *p* = 0.027, mid-anterior, *r*(29) = -0.441, *p* = 0.027, right-anterior, *r*(29) = -0.495, *p* = 0.018, mid-central, *r*(29) = -0.449, *p* = 0.019, and right-central, *r*(29) = -0.467, *p* = 0.018 (see Figure 3), whereas the correlation was not significant in the time window of 200–400 ms (all *p*s > 0.5).

**FIGURE 4:**
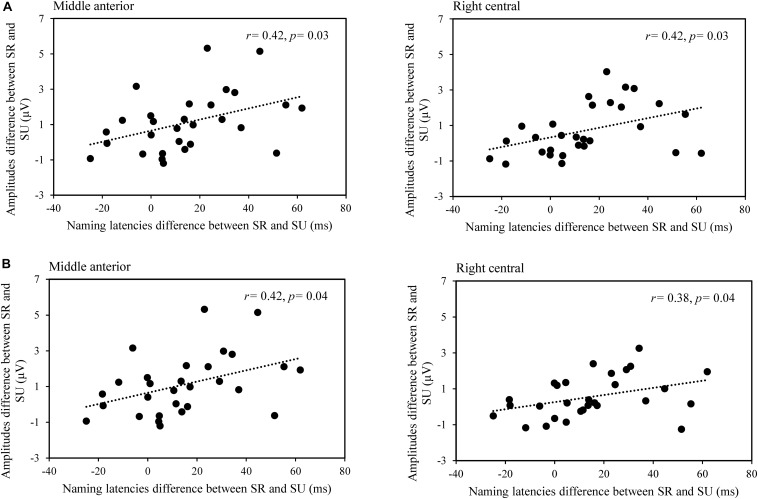
The scatterplot for the correlation between RT (semantically related minus unrelated) and the mean amplitudes (semantically related minus unrelated) in middle anterior and right central regions in the time windows of 200–400 ms **(A)** and 400–600 ms **(B)**.

We also performed a correlation analysis between the difference of naming latencies and difference of averaged amplitude for semantically related and unrelated conditions across nine ROIs in the time windows of 200–400 and 400–600 ms, respectively. Results showed positive correlations in the mid-anterior, *r*(29) = 0.416, *p* = 0.025, right-anterior, *r*(29) = 0.419, *p* = 0.025, and right-central regions, *r*(29) = 0.418, *p* = 0.025 in the time window of 200–400 ms (see Figure 4A), and they showed positive correlations in the mid-anterior, *r*(29) = 0.416, *p* = 0.042, right-central regions, *r*(29) = 0.379, *p* = 0.042 in the time window of 400–600 ms (see Figure 4B). Overall, the findings above indicated that lexical cohort size produces facilitation in the time window of 100–200 ms, and semantic relatedness produces semantic interference in the time windows of 200–400 and 400–600 ms.

### sLORETA Analysis

**FIGURE 5:**
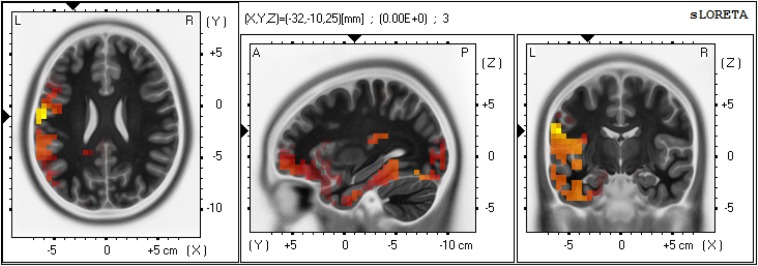
Three-dimensional maps and associated scalp maps between large and lexical cohort sizes in the time window of 250–300 ms using sLORETA.

The result of sLORETA statistical non-parametric maps showed a significant difference between large and small lexical cohort sizes in the time window of 250–300 ms. Especially when compared to the small lexical cohort condition, the large lexical cohort condition showed greater activation in the left STG (BA 22; MNI coordinate: *X* = -65, *Y* = 20, *Z* = 0) (Log-*F*-ratio = 1.29, *p* = 0.03) and IFG (BA 9; MNI coordinate: *X* = 45, *Y* = 0, *Z* = 35) (Log-*F*-ratio = 1.36, *p* = 0.02). Figure 5 presents the three-dimensional maps for the lexical cohort size effect with low resolution electromagnetic tomography. There was no significant difference between semantically related and unrelated conditions in any cerebral region during any of the time frames.

## Discussion

Using a PWI task combined with ERP and sLORETA analyses, we investigated whether lexical cohort size impacts semantic context effects in spoken word production. Behavioral results showed the main effects of lexical cohort size and semantic relatedness but no interaction between them, and these results were in line with previous studies (Vigliocco et al., 2002; Mirman, 2011; Vieth et al., 2014; Rabovsky et al., 2016). In the PWI task, the production system receives activation from targets and distractors, and both sources influence each other. On one hand, targets from categories with larger lexical cohorts theoretically produce stronger semantic activation than those from categories with small lexical cohorts at the conceptual level and, accordingly, stronger lexical activation at the subsequent lexical level. This was confirmed by shorter naming latencies in larger lexical cohorts in comparison with small lexical cohorts. On the other hand, distractors from larger lexical cohorts theoretically produce stronger interference than those from small lexical cohorts, which was also confirmed by stronger semantic interference effects in larger lexical cohorts.

Event-related potential results revealed lexical cohort size effect in the time windows of 100–200 and 200–400 ms and semantic context effects in the time windows of 200–400 and 4007–600 ms. In accordance with behavioral results, there was no interaction between semantic relatedness and lexical cohort size. Furthermore, behavior-ERP correlation analysis revealed that the decreased average amplitude elicited by large lexical cohorts correlated with faster picture naming in the time window of 100–200 ms, and increased average amplitude elicited by semantically related distractors correlated with slower picture naming in the time windows of 200–400 and 400–600 ms, suggesting the presence of an early facilitation effect at the conceptual level and a later interference effect at the lexical level. Additionally, sLORETA analysis indicated that the lexical cohort size effect in the time window of 250–300 ms was associated with greater activation in the left STG and IFG, which closely related to lexical selection in spoken word production (Schnur et al., 2009; Dhooge and Hartsuiker, 2012; Mirman and Graziano, 2013).

### The Lexical Cohort Size Effect in the Time Windows of 100–200 and 200–400 ms

An important finding was that the lexical cohort size effect in the time window of 100–200 ms mainly in frontal and central regions, with the target pictures from large cohorts eliciting a more positive-going wave than those from small cohorts (see Figure 1C). Modulations in this time window reflected semantic processing at the conceptual level (Indefrey, 2011; Rose and Abdel Rahman, 2017), and the activation of picture names from large cohorts was higher than those from small cohorts, which was in line with the SLN. A possible cognitive mechanism that underlies the lexical cohort size effect is the activation of a semantic cohort comprised of semantically related concepts. Roelofs (1992) proposed that when a concept (e.g., dog) is processed, not only the target but also categorically related concepts (e.g., cat, horse, and tiger) are activated. These concepts will naturally induce a semantic cohort through activation at a superordinate level, which is sensitive to the number of category members (Bloem et al., 2004).

Further correlation analysis showed that the averaged amplitude difference between large and small lexical cohorts was negatively correlated with differences in naming latencies between them, suggesting that the semantic context effect is facilitative at the conceptual level (see Figure 3). The increased amplitude elicited by targets with larger cohorts relative to ones with small cohorts may reflect the increased activation of semantic features shared by more category members (Abdel Rahman and Sommer, 2008; Dell’Acqua et al., 2010; Rose and Abdel Rahman, 2017) or attention allocation (Hunt and Seta, 1984). Hunt and Seta (1984) have demonstrated that attention is more likely to be focused when a large number of exemplars share a set of semantic features.

Our results were in line with the studies using the semantic blocking paradigm, and this demonstrated that the activation of the semantic system was susceptible to dynamic changes in specific situations (Vigliocco et al., 2002; Abdel Rahman and Melinger, 2011). In these studies, pictures were presented and named in blocks either comprising objects from the same semantic category or objects from different semantic categories. This paradigm created a discourse context in which related concepts activated each other; as a result, the semantic activation level of concepts, including the previously named category members, could be strengthened. Notably, presenting objects with an isolated distractor word like the present study also constructed a special semantic context (i.e., categorical relation) in which the related concepts were automatically activated in terms of the given category. The current study extended previous findings to the PWI paradigm, implying that the semantic cohort activated by categorical relations was also highly flexible and capable of adjusting to suit semantic contexts.

Interestingly, we also found a lexical cohort size effect distributed over the left-frontal and central regions in the time window of 200–400 ms; there were more negative waveforms for targets from large cohorts (see Figures 1C, 2C). There is an increasing amount of evidence to support the fact that this time window is crucial for lexical selection (Costa et al., 2009; Dell’Acqua et al., 2010; Indefrey, 2011; Piai et al., 2012; Zhu et al., 2015; Rose and Abdel Rahman, 2017; Wong et al., 2017). We therefore speculate that the lexical cohort size effect on mean amplitudes may reflect the number of co-activated lexical representations. The targets with large lexical cohorts automatically spread activation to semantically related concepts and naturally produce larger sizes of semantic cohorts than those with small cohorts. In the WEAVER model, the activation spread between conceptual and lexical layers is bidirectional, which is fundamental to the occurrence of semantic context effects (Levelt et al., 1999). Given the assumption that all activated concepts could automatically activate their corresponding lexical representations (Roelofs, 1992), targets with large cohorts would co-activate more lexical representations than those with small cohorts. According to the SLN, more activated lemmas would theoretically make lexical selection of the target more difficult, consequently resulting in longer naming latencies.

However, we did not find a significant correlation between the difference of ERP-averaged amplitudes (large minus small) and difference of naming latencies (large minus small) in the time window 200–400 ms. One possibility is that there exists a trade-off between the facilitation effect at the conceptual level and the interference effect at the lexical level, which renders the relative activation levels unspecified. Mahon and Caramazza (2009) proposed that there could be low-level activation in more items in a large lexical cohort and relatively high-level activation in fewer items in a small lexical cohort. By contrast, Chen and Mirman (2012) suggested that strongly active neighbors exert a net interference effect, and weakly activated neighbors exert a net facilitative effect. Fieder et al. (2018), however, found that the lexical cohort size (i.e., semantic neighborhood density) has a detrimental effect on picture naming for only the close semantic neighbors but not for the distant or the category-specific semantic neighbors. Different semantic contexts in different paradigms produce distinct results, and we thus suggest that the activation of the lexical cohort is flexible and dynamic to the message-inherent semantic attributes and the context in which the specific items are created during the experiment (see also Mirman, 2011). Note that naming latencies are influenced by many different processes; facilitation and interference effects may be entirely unrelated to the difference in mean amplitudes between large and small cohorts. Thus, the interference related to lexical selection might be undetectable in the correlation analysis (see Rose and Abdel Rahman for a similar account).

### The Semantic Interference Effect in the Time Window of 200–400 ms

A semantic effect was found in the time window of 200–400 ms, with a more positive-going waveform for semantically related distractors than unrelated ones, and this different activity was widely distributed over frontal and central regions (see Figures 1, 2). The time course and topographical distribution of the semantic effect was in agreement with other studies using different paradigms (Costa et al., 2009; Dell’Acqua et al., 2010; Piai et al., 2012; Zhu et al., 2015; Rose and Abdel Rahman, 2017; Wong et al., 2017) and a recent meta-analysis study (Indefrey, 2011). Using a PWI paradigm and Mandarin materials, Zhu et al. (2015) observed that Mandarin speakers produced a semantic interference effect in a similar time window of 250–450 ms. Consistent with these studies, our correlation analysis revealed a significant positive correlation in differences between ERP amplitudes (the frontal and central regions) and picture-naming latencies, reflecting that increased difficulty in lemma selection is associated with increased naming latencies for semantically related distractors in comparison to unrelated ones (see Figure 4A). We therefore suggest that the semantic effect in this time window reflects the competition of all co-activated lexical entries with target lemma, and a more negative-going waveform indicates stronger competition for semantically related distractors in comparison to unrelated items.

The absence of interaction between lexical cohort size and semantic relatedness was inconsistent with the SLN (Abdel Rahman and Melinger, 2009b), but it was in line with Bormann’s (2011), study (2011, experiment 3) in the PWI task. We suggest that lexical cohort size is independent of semantic relatedness in spoken word production (see also Bormann, 2011). Given that the factor of lexical cohort size is the property of target pictures, while the factor of semantic relatedness is the property of distractor words, it is possible that two independent sources of activation influence picture naming independently and do not interact in the production process (Bormann, 2011). Another possibility is that there is trade-off between facilitation and interference resulting from the lexical cohort size, and the net activation was probably weaker than the semantic interference effect resulting from distractors. In other words, lexical cohort size has little effect on lexical selection competition because the co-activated lexical representations are not activated enough to constrain the target lexical selection except for the visually presented semantic-related distractor. This argument is supported by Rose and Abdel Rahman’s (2017) study (2017), in which they successfully disassociated the factors of lexical activation level and lexical cohort size, and they found that an increase in lexical cohort size does not necessarily lead to an increase in lexical activation.

Furthermore, the current study observed that the ERP modulations induced by large lexical cohorts were more negative going, while the modulations elicited by semantically related distractors were more positive going. This might suggest that mechanisms underlying lexical cohort size and semantic relatedness should be distinct, with the former reflecting the number of lexical representations co-activated with targets and the latter reflecting the lexical competition for selection. However, considering that this study is the first ERP investigation into lexical cohort size, more studies are needed in the future to enrich our understanding of its underlying mechanism.

Some studies found that the interaction between conceptual and lexical levels for categorical relations were consonant with the SLN (Abdel Rahman and Melinger, 2011; Aristei et al., 2011). Aristei et al. (2011) combined the PWI paradigm and semantic blocking paradigm to investigate the temporal courses of semantic facilitation and interference effects. Pictures were categorically homogeneous objects, associatively homogeneous objects, or heterogeneous (categorically and associatively unrelated) objects, and each picture was paired with three auditory distractor words, which were categorically related, associatively related, or unrelated to the picture name. They found that there was an interaction between the semantic blocking context and semantic relatedness in both RTs and ERPs. For the RT results, the interaction was mainly from associative but not categorical relations. Our study also addressed categorical relations, and the behavioral finding was in line with the finding of categorical relations in Aristei et al. (2011) study. For the ERP results, categorical relations produced a weaker and short-interval interaction (250–300 ms after pictures onset) in Aristei et al. (2011) study. They proposed that a categorically related distractor word could not produce strong interactions with a cohort if the cohort was activated by the PWI manipulation alone. In the present study, we manipulated the lexical cohort size and categorical relation between distractor and target. The activation of a cohort originates from two sources: a categorically related distractor and targets belonging to a category. However, since targets within a category were not presented in consecutive trials, the activation of the cohort from targets would be low. We therefore suggest that, in the PWI task, the activation of cohorts is probably too weak to produce a significant interaction of two variables at the lexical level. Taken together, these findings demonstrate that the semantic relation between conceptual and lexical activation is sensitive to dynamic adaptations modulated by attention, intentions, and situations.

### The Semantic Interference Effect in the Time Window of 400–600 ms

We also observed a later semantic effect in the time window of 400–600 ms, with semantically related distractors eliciting larger frontal and central positive-going waveforms compared to unrelated items (see Figures 1, 2), which replicates previous studies that used a similar task (Roelofs and Piai, 2015; Wong et al., 2017). Is it possible that this effect localized at the post-lexical level as the response exclusion hypothesis predicted? Dhooge et al. (2013) reported distractor frequency effects in the time windows of 420–500 and 520–580 ms, and they attributed these ERP effects to the post-lexical level rather than to the lexical level. However, Roelofs and Piai (2015) suggested that the distractor frequency effects do not occur earlier than about 145 ms before articulation onset. As the averaged naming latencies (i.e., 780 ms) in Dhooge et al. (2013) were much longer than an average of 600 ms in a meta-analysis (Indefrey and Levelt, 2004), it is likely that these ERP effects (420–580 ms) might occur in the process of word retrieval (i.e., 260–592 or 380–635 ms) rather than in the process of articulatory buffering (after 592 or 635 ms).

On the other hand, studies demonstrated that the semantic effect in this time window was related to processes at the lexical level in spoken word production. Wong et al. (2017) observed categorical interference effects in the time window of 275–450 and 450–600 ms, and they interpreted both effects as being associated with lexical selection competition based on a response-locked analysis. The semantic interference effect observed in this time window has been interpreted as the processing of lexical-semantic encoding, and the origin of the effect has been thought to be stem from the interface of the conceptual and the lexical level (Belke, 2013; Belke and Stielow, 2013; Rose and Abdel Rahman, 2017). For example, using a continuous naming task, Rose and Abdel Rahman (2017) observed semantic interference on the ERPs signals in the time window of 450–600 ms, reflecting a semantic-lexical calibration process.

Given that naming latencies (900 ms) here were longer than the average (i.e., 600 ms) in a meta-analysis (i.e., Indefrey, 2011) and our stimuli comprised several exemplars belonging to the same categories, the semantic interference effect we observed may be reflective of the cumulative semantic effect, which is similar to the long-lasting effect observed in the continuous naming task (Scaltritti et al., 2017). Furthermore, we found similar correlation patterns between naming latencies and the mean amplitude in both time windows, and we therefore suggest that the semantic context effects in the time window of 200–400 and 400–600 ms were associated with lexical selection by competition and not the response-output buffer assumed by the response-exclusion hypothesis.

### Source Estimation of Lexical Cohort Size Effect

Standardized low-resolution brain electromagnetic tomography comparisons of the lexical cohort size revealed more current density in the left STG and IFG for large lexical cohorts compared with small ones. A number of neuroimaging studies suggested that the left STG is responsible for lexical selection (Hocking et al., 2009) or internal self-monitoring activity (McGuire et al., 1996; Indefrey and Levelt, 2000; Indefrey, 2011). For example, using the semantic blocking paradigm in a fMRI study, Hocking et al. (2009) observed STG activation in the semantic homogeneous condition, reflecting increased demands on lexical selection and self-monitoring. A meta-analysis conducted by Indefrey (2011) suggested a time window for lexical selection between 200 and 350 ms, during which co-activated lemmas compete with target for selection. The increased burdens call for the greater involvement of the self-monitoring system (see also Ganushchak and Schiller, 2008), and this consequently leads to higher activation levels in the left STG. Furthermore, the left IFG has also been involved in lexical selection among active competitors (Mirman and Graziano, 2013). Thus, the greater activation of left STG and IFG for targets from large lexical cohorts may help select target lemma among more co-activated competitors.

To summarize, using a PWI paradigm, we first observed that lexical cohort size affected speech production at the conceptual and lexical levels. More importantly, there was no interaction between lexical cohort size and semantic relatedness for categorical relations. Our findings provide evidence for the SLN rather than the response-exclusion hypothesis. Future research should investigate the relation between conceptual and lexical activation with different semantic relations and different paradigms.

## Data Availability Statement

All datasets generated for this study are included in the article/Supplementary Material.

## Ethics Statement

This study was approved by the ethics board of the Renmin University of China, as well as by the participating schools. The patients/participants provided their written informed consent to participate in this study. Written informed consent was obtained from the individual(s) for the publication of any potentially identifiable images or data included in this article.

## Author Contributions

MO and QZ designed the study. MO conducted the experiment and prepared the manuscript. XC performed the data analysis and manuscript revision. QZ supervised the whole research and wrote the manuscript.

## Conflict of Interest

The authors declare that the research was conducted in the absence of any commercial or financial relationships that could be construed as a potential conflict of interest.

## Footnotes

1 Results indicated that there was no interaction between repetition and cohort size, reflecting that the lexical cohort size effect was not influenced by repetition of targets and distractors but arose online in the production process.
