# Supplementary material for: The Effect of Lexical Cohort Size Is Independent of Semantic Context Effects in a Picture–Word Interference Task: A Combined ERP and sLORETA Study
Source: Front Hum Neurosci. 2019 Dec 20;13:439. doi: 10.3389/fnhum.2019.00439 (PMC6933526; doi:10.3389/fnhum.2019.00439)
Supplement: Supplementary file 1 [file Table_1.docx]

| Appendix A: Stimuli used in experiment. | | | | | | | |
| --- | --- | --- | --- | --- | --- | --- | --- |
| Large Categories | | | | Small Categories | | | |
| Category Names | Target Names | Semantically Related | Semantically Unrelated | Category Names | Target Names | Semantically Related | Semantically Unrelated |
| 哺乳动物/Mammal | 母牛/Cow | 熊猫/Panda | 尺子/Ruler | 人体器官/Body organ | 肩膀/Shoulder | 拇指/Thumb | 帆船/Sailing boat |
|  | 大象/Elephant | 狮子/Lion | 毛巾/Towel |  | 胳膊/Arm | 耳朵/Ear | 鼠标/Mouse |
|  | 兔子/Rabbit | 老虎/Tiger | 铅笔/Pencil |  | 舌头/Tongue | 膝盖/Knee | 报纸/Newspaper |
|  | 绵羊/Sheep | 老鼠/Rat | 项链/Necklace |  | 牙/Tooth | 肾/Kidney | 桶/Barrel |
|  | 猪/Pig | 狗/Dog | 鞋/Shoes |  | 鼻/Nose | 肺/Lungs | 锚/Anchor |
|  | 猴子/Monkey | 骆驼/Camel | 花篮/Basket |  | 脑/Brain | 胃/Stomach | 弓/Bow |
| 鸟类/[Bird](C:/Users/Administrator/AppData/Local/youdao/dict/Application/6.3.69.8341/resultui/frame/javascript:void(0);) | 鸽子/Dove | 麻雀/Sparrow | 橡皮/Rubber | 乐器/ Instrument | 钢琴/Piano | 喇叭/Horn | 陀螺/Top |
|  | 公鸡/Cock | 喜鹊/Magpie | 火箭/Rocket |  | 吉他/Guitar | 口琴/Harmonica | 牙膏/Toothpaste |
|  | 老鹰/Eagle | 乌鸦/Crow | 地图/Map |  | 提琴/Violin | 琵琶/Pipa | 酒杯/Wineglass |
|  | 孔雀/Peacock | 大雁/Goose | 剪刀/Scissors |  | 竖琴/Harp | 古筝/Zither | 螺丝/Screw |
|  | 鹦鹉/Parrot | 海鸥/Sea gull | 排球/Volleyball |  | 小号/Trumpet | 二胡/Erhu | 拉链/Zipper |
|  | 燕子/Swallow | 鸵鸟/Ostrich | 皮带/Belt |  | 长笛/Flute | 扬琴/Dulcimer | 手表/Watch |
| 水果/Fruit | 菠萝/Pineapple | 荔枝/Litchi | 拐杖/Cane | 家具/Furniture | 桌子/Desk | 立柜/Dresser | 风车/Windmill |
|  | 香蕉/Banana | 西瓜/Watermelon | 扑克/Poker |  | 凳子/Stool | 壁橱/Closet | 肥皂/Soap |
|  | 草莓/Strawberry | 樱桃/Cherry | 钟表/Clock |  | 衣柜/Wardrobe | 书架/Bookshelves | 轮胎/Tire |
|  | 橘子/Orange | 桃子/Peach | 烟囱/Chimney |  | 椅子/Chair | 花架/Flower stand | 戒指/Ring |
|  | 苹果/Apple | 柠檬/Lemon | 皇冠/Crown |  | 茶几/Table | 板凳/Bench | 雪人/Snowman |
|  | 葡萄/Grape | 石榴/Granada | 信封/Envelope |  | 沙发/Sofa | 床架/Bedstead | 雨伞/Umbrella |
| 蔬菜/Vegetable | 南瓜/Pumpkin | 大豆/Soybean | 头盔/Helmet | 家电/Appliance | 洗衣机/Washing machine | 收音机/Radio | 拖拉机/Tractor |
|  | 青椒/Pepper | 油菜/Rape | 喷泉/Fountain |  | 冰箱/Refrigerator | 烤炉/Oven | 酒瓶/Bottle |
|  | 茄子/Eggplant | 花生/Peanut | 手铐/Handcuffs |  | 电扇/Fan | 唱机/Gramophone | 火柴/Match |
|  | 黄瓜/Cucumber | 土豆/Potato | 钥匙/Key |  | 电筒/Flashlight | 音箱/Loudspeaker | 手铐/Handcuffs |
|  | 萝卜/Turnip | 玉米/Corn | 黑板/Blackboard |  | 熨斗/Iron | 吹风机/Hairdryer | 栅栏/Fence |
|  | 白菜/Cabbage | 芹菜/Celery | 衣架/Hanger |  | 电饭煲/Cooker | 录音机/Recorder | 三明治/Sandwich |
